# Anti-inflammatory effects of anemonin on acute ulcerative colitis via targeted regulation of protein kinase C-θ

**DOI:** 10.1186/s13020-022-00599-3

**Published:** 2022-03-28

**Authors:** Lu Jiang, Chunhua Chi, Fang Yuan, Meiqi Lu, Dongqing Hu, Lin Wang, Xiaoming Liu

**Affiliations:** 1 Department of Gastroenterology, Affiliated Hospital of Shandong University of Traditional Chinese Medicine, No.42 Wenhua west road, Jinan, 250011 Shandong China; 2 Department of Anorectal Surgery, Affiliated Hospital of Shandong University of Traditional Chinese Medicine, Jinan, 250011 Shandong China; 3 Department of Gastrology, Affiliated Hospital of Shandong University of Traditional Chinese Medicine, Jinan, 250011 Shandong China; 4 Affiliated Hospital of Shandong University of Traditional Chinese Medicine, Jinan, 250011 Shandong China; 5 Department of Geriatrics, Affiliated Hospital of Shandong University of Traditional Chinese Medicine, No.42 Wenhua west road, Jinan, 250011 Shandong China

**Keywords:** Anemonin, Acute ulcerative colitis, Inflammation, PKC-θ

## Abstract

**Background:**

Ulcerative colitis (UC) is an inflammatory bowel disease that causes continuous mucosal inflammation. Anemonin is a natural molecule from the Ranunculaceae and Gramineae plants that exerts anti-inflammatory properties. This study aimed to explore the effects and mechanisms of anemonin on UC.

**Methods:**

C57BL/6 mice were administered dextran sulphate sodium (DSS; 3% [w/v]) to establish an animal model of UC. Mice were treated with an intraperitoneal injection of anemonin. Body weight and the disease activity index (DAI) were recorded. Haematoxylin and eosin staining, RT-qPCR, ELISA, and western blotting were performed to evaluate the histopathological changes and tissue inflammation. HT-29 cells were treated with lipopolysaccharide (LPS) and anemonin. Cell inflammation was evaluated using RT-qPCR and western blotting. The target proteins of anemonin were predicted using bioinformatics analysis and confirmed in vitro and in vivo.

**Results:**

Anemonin improved DSS-induced body weight loss, shortened colon length, increased DAI, and induced pathological changes in the colon tissue of mice. Anemonin inhibited DSS-induced colon tissue inflammation as the release of IL-1β, TNF-α, and IL-6 was significantly suppressed. Additionally, anemonin attenuated LPS-induced cytokine production in HT-29 cells. PKC-θ was predicted as a target protein of anemonin. Anemonin did not affect PRKCQ gene transcription, but inhibited its translation. PRKCQ overexpression partially reversed the protective effects of anemonin on HT-29 cells. Adeno-associated virus delivery of the PRKCQ vector significantly reversed the protective effects of anemonin on the mouse colon.

**Conclusions:**

Anemonin has the potential to treat UC. The anti-inflammatory effects of anemonin may be mediated through targeting PKC-θ.

## Background

Ulcerative colitis (UC) is an inflammatory bowel disease (IBD) of the colon that causes continuous mucosal inflammation. Its main clinical manifestations are abdominal pain, diarrhoea, and mucous blood stools, which are always accompanied by weight loss, tenesmus, vomiting and other symptoms. UC is characterized by recurrent inflammation of the colorectum with ulceration of the intestinal mucosa [1]. Histologically, UC primarily invades the mucosa and submucosa of the colorectum. It is generally believed that during UC, various immune cells, such as myeloid cells and lymphocytes, accumulate and contribute to the immune response, and intestinal epithelial cells are damaged. Owing to the production of these immune cells and the inflammatory mediators secreted by UC, intestinal inflammation in UC persists [2].

Currently, the clinical treatment of UC includes drug and surgical therapies, and therapeutic drugs include aminosalicylic acids, glucocorticoids, immunosuppressants, and traditional Chinese medicine preparations [3]. These drugs can effectively inhibit intestinal inflammation and mucosal barrier dysfunction during UC pathogenesis. However, the numerous adverse reactions, high incidence, and side effects of long-term application still limit the use of these drugs [4]. Recently, with increasing studies on the relationship between intestinal flora and UC, researchers have attempted to use flora transplantation to treat UC, and found that it can significantly improve the clinical remission rate of patients [5]. However, the pathophysiological changes brought about by intestinal flora transplantation in UC patients require long-term observation [6]. Therefore, it is of considerable significance to identify new therapeutic targets for the prevention and treatment of UC.

Recent studies indicate that the occurrence of UC is closely related to immune function, intestinal microbes, genetic factors and environmental factors, among which immune dysfunction plays a crucial role in the occurrence and development of UC [7]. Cytokines are small molecular proteins with certain biological activities generated by stimulated immune or non-immune cells. They participate in the regulation of cell growth, differentiation, immune response, and other functions, and are important mediators of mutual regulation between cells, forming a huge signal network [8]. Understanding cytokine networks in the gut has laid the foundation for the development of multiple biological therapies for IBD treatment [9]. Interleukin (IL)-6 can induce the transformation of UC into chronic inflammation [10]. Dysregulation of TGF-β signaling has been observed in the intestinal tract of patients with IBD [11]. The establishment of mouse models has revealed an important role of IL-10 in maintaining intestinal homeostasis [12].

Anemonin exists in various Ranunculaceae and Gramineae plants, has a variety of biological activities such as antitumour, antibacterial, analgesic, and sedative properties, and has been widely used in the study of several diseases [13, 14]. Anemonin can inhibit the release of IL-6 from mouse peritoneal macrophages induced by endotoxins, alleviate excessive inflammatory responses and systemic injury, and has a good therapeutic effect on endotoxaemia [15]. Furthermore, inhibition of nitric oxide production by lipopolysaccharide (LPS)-induced endothelial cells, has been shown to have anti-inflammatory effects [16].

Based on the above research, we demonstrated the alleviating effect of anemonin in acute UC. To test this hypothesis, we used a mouse model and human colon cells as experimental subjects to identify whether anemonin can alleviate the symptoms of acute UC caused by dextran sulphate sodium (DSS). In a cellular model, anemonin was tested to determine whether it significantly alleviated LPS-induced inflammation. Through bioinformatics, the target protein of anemonin was identified and verified to explore its mechanism of action, providing theoretical support for seeking new drug therapeutic targets for the treatment of UC.

## Methods

### Animals

C57BL/6 J mice were purchased from Jinan Pengyue Laboratory Animal Co., Ltd. (Jinan, China) and were provided unlimited access to food and water throughout the study. The circadian rhythm was 12 h in light (8:00 am–8:00 pm) and 12 h in the dark. Relative humidity was 55 ± 5%, and the temperature was maintained at 22 ± 2 °C. The animal study protocol was approved by the Animal Care and Use Committee of the Affiliated Hospital of Shandong University of Traditional Chinese Medicine.

An acute UC mouse model was induced with DSS. The DSS concentration in the drinking water of the model group mice was 3% [w/v], and the subsequent experiment was conducted after continuous feeding of this concentration solution for 7 days. To detect the inhibitory effect of anemonin on UC, different anemonin concentrations (2, 5, and 10 mg/kg) were intraperitoneally injected into the mice during the establishment of the DSS-induced UC model. The general condition of the mice was assessed using the disease activity index (DAI) using the following formula: DAI = (weight loss [%] + characterization of feces + hematochezia)/3[17].

### Adeno-associated virus (AAV) vector infection

AAV vectors carrying PRKCQ were purchased from Genechem Co., Ltd. (Shanghai, China). AAV vectors expressing no transgenes served as the negative control (AAV-null). One week before DSS administration, the mice were anesthetized using 3% pentobarbital sodium, and a soft catheter was inserted into the mouse anus at a depth of 4 cm. Sterile saline containing 0.2 mL of AAV-PRKCQ or AAV-null (4 × 10^10^ viral genome) was instilled into the mouse colon via a catheter. The mice were placed upside down for 1 min to allow the enema to be distributed throughout the colon [18].

### Haematoxylin–eosin (HE) staining

HE staining was performed using a Haematoxylin–eosin Staining Kit (cat. no.C0105S, Beyotime, China). Briefly, after dewaxing, the slices were incubated with haematoxylin for 2 min and stained with eosin. Dehydrated slices were then embedded for microscopic analysis.

### Enzyme-linked immunosorbent assay (ELISA)

IL-1β, IL-6, and tumour necrosis factor-alpha (TNF-α) levels were measured using ELISA kits (Elabscience, Wuhan, China) according to the manufacturer's instructions. Optical density was measured at 450 mm using a Varioskan LUX microplate reader (Thermo Fisher Scientific Inc., MA, USA) in each well.

### HT-29 cell culture

HT-29 cells (Procell, Wuhan, China) were cultured in Dulbecco’s modified Eagle medium (cat. no. 11965092, Thermo Fisher Scientific, USA) with 10% foetal bovine serum (FBS; cat. no. 30067334, Thermo Fisher Scientific), and 1% streptomycin/penicillin (cat. no. 15070063, Thermo Fisher Scientific) and maintained at 37 °C in an atmosphere of 5% CO_2_.

The cells were treated with LPS to establish an inflammatory cell model. Cells (5 × 10^4^ cells/mL) were inoculated into 96-well plates (100 μL per well). After culturing for 24 h, the cells were treated with LPS (1 μg/mL) and cultured for 24 h before the subsequent experiments. HT-29 cells were treated with different anemonin concentrations (2.5, 5, and 10 μM) for 48 h.

### Cell transfection

Lipofectamine 2000 reagent (cat. no. 11668030, Thermo Fisher Scientific) was used for cell transfection according to the manufacturer’s instructions. Briefly, HT-29 cells were seeded at 1.5 × 10^5^ cells/cm^2^. The vectors were mixed with Lipofectamine 2000 for 20 min at room temperature to form the Lipo2000/DNA complex. Next, 100 μL of the complex was gradually dripped into the culture solution and mixed with coculture for 4–6 h. The complex was discarded, and the culture medium was replaced with a medium containing 1% streptomycin/penicillin and 10% FBS for 24 h before subsequent experiments.

### Flow cytometry

An annexin V-FITC apoptosis detection kit (cat. no. C1062S; Beyotime) was used to detect HT-29 cell apoptosis. Flow cytometry (BD Biosciences, CA, USA) was used to determine the percentage of apoptotic cells.

### RT-qPCR

Total RNA was extracted from colon tissues and HT-29 cells using TRIzol reagent and then reverse-transcribed into DNA using an RT-PCR Kit (#K1622, Thermo Fisher Scientific) according to the manufacturer's instructions. Real-time PCR was performed with the SYBR Green PCR kit (F-415XL, Thermo Fisher Scientific) on a Real-Time PCR System (ABI-7500, Applied Biosystems, MA, USA) using the GAPDH gene as an internal control [19]. The 2-ΔΔCt method was used to analyze the data [20]. Primers used for qPCR were purchased from Sangon Biotech (Shanghai, China). Primers were designed using the Primer-BLAST tool [21].

### Western blotting

Proteins were extracted using RIPA total protein lysate (cat. no. P0013C, Beyotime) according to the manufacturer's instructions. Proteins from each sample were separated by electrophoresis on 10% SDS-PAGE gels and transferred onto PVDF membranes (cat. no. FFP39, Beyotime). After incubation with the blocking solution (cat. no. P0252, Beyotime) for 1 h at room temperature, the PVDF membranes were separately incubated with primary antibodies (1:1000; Abcam, Cambridge, UK) at 4 °C overnight. After the blots were washed, they were incubated with HRP-conjugated goat anti-rabbit IgG H&L secondary antibody (1:2000; cat.no.ab6721, Abcam) for 1.5 h and detected using ECL western reagent (cat. no. P0018S, Beyotime) on a chemiluminescent imaging system (ChemiDoc MP, Bio-Rad, CA, USA).

### Bioinformatic analysis

SwissTargetPrediction was used to predict the target proteins of anemonin. The Comparative Toxicogenomics Database (CTD) was used to predict the genes associated with UC. The intersection of these two results was predicted to be a potential anemonin target protein. AutoDock Vina software was used to dock anemonin with a potential target protein, and the binding free energy was calculated. Discovery Studio 4.5 Client software was used to further analyze the interaction results.

### Statistical analysis

Statistics and mapping were performed using GraghPad Prism software. One-way ANOVA analysis followed by Tukey’s post-hoc test was used to analyze the differences between groups. The data were considered significantly different at *p* < 0.05.

## Results

### Anemonin improves DSS-induced acute UC in mice

**Fig. 1:**
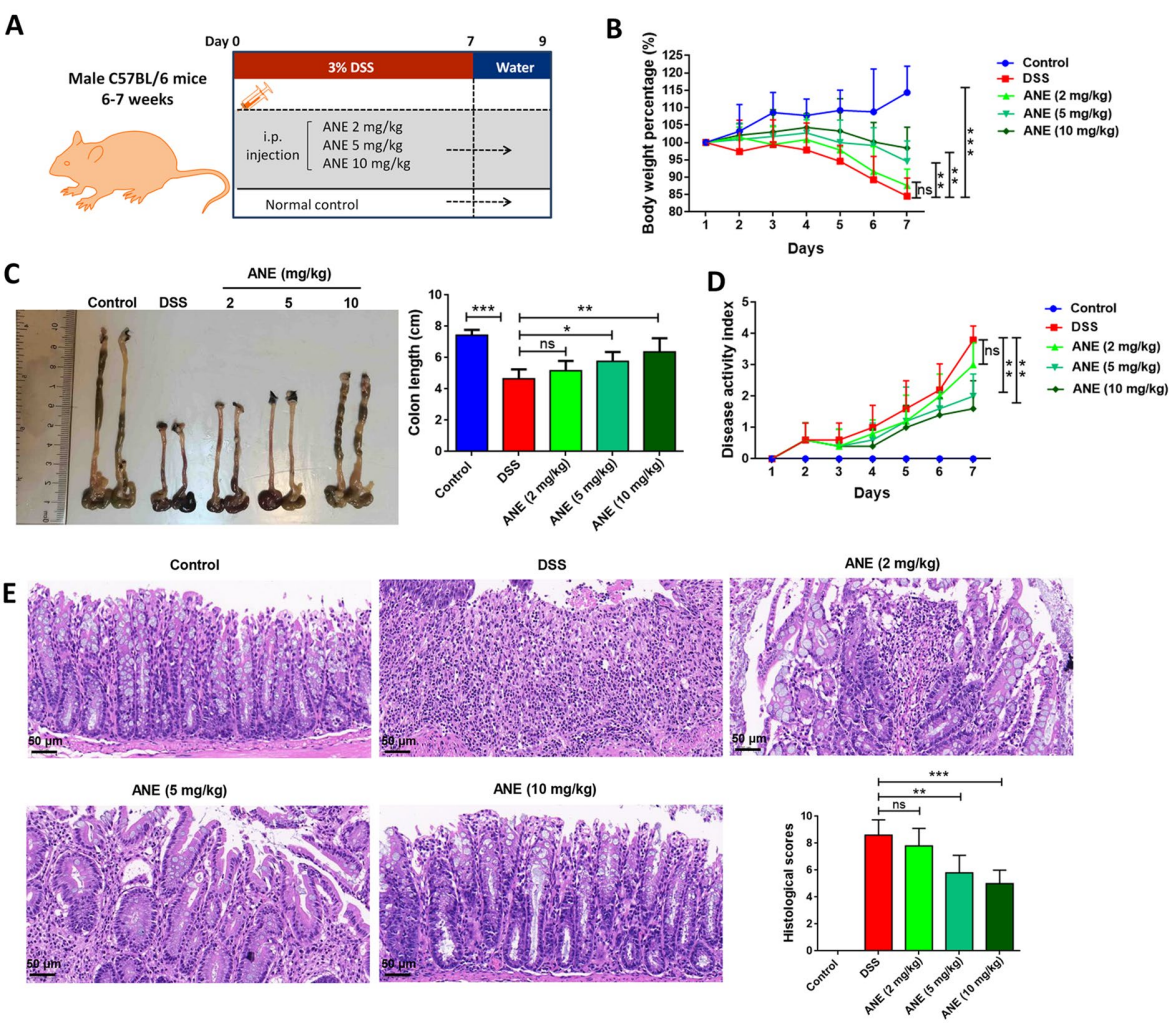
Anemonin improves DSS-induced acute UC in mice. **A** Mouse model and schematic diagram of drug action. **B** Daily weight change of mice. **C** Colon length statistics at the end of the experiment. **D** Disease activity was assessed by DAI score. **E** Colonic tissues of mice were stained with HE staining to evaluate the degree of tissue damage. *ns* no significance, *p < 0.05,**p < 0.01, ***p < 0.001

DSS was used to establish a mouse UC model to identify the pharmacological effects of anemonin in UC. Male C57BL/6 mice aged 6–7 weeks were used as research subjects, and the model group was provided water containing 3% DSS daily for 7 days (Fig. 1A). The drug group received daily DSS and intraperitoneal injection of different anemonin doses. Sterile water was used for the control group. After 7 days, all mice were administered sterile water and sacrificed on day 9 for follow-up experiments. As shown in Fig. 1B and C, anemonin prevents weight loss and colon length shortening in DSS-induced UC mice in a dose-dependent manner (*p* < 0.05). DSS significantly increased the DAI score, whereas anemonin significantly inhibited the DAI score in a dose-dependent manner (*p* < 0.05, Fig. 1D). HE staining results showed that the histopathological changes in colon tissues induced by DSS were significantly attenuated by anemonin in a dose-dependent manner (*p* < 0.05, Fig. 1E).

### Anemonin improves DSS-induced inflammation in mice

**Fig. 2:**
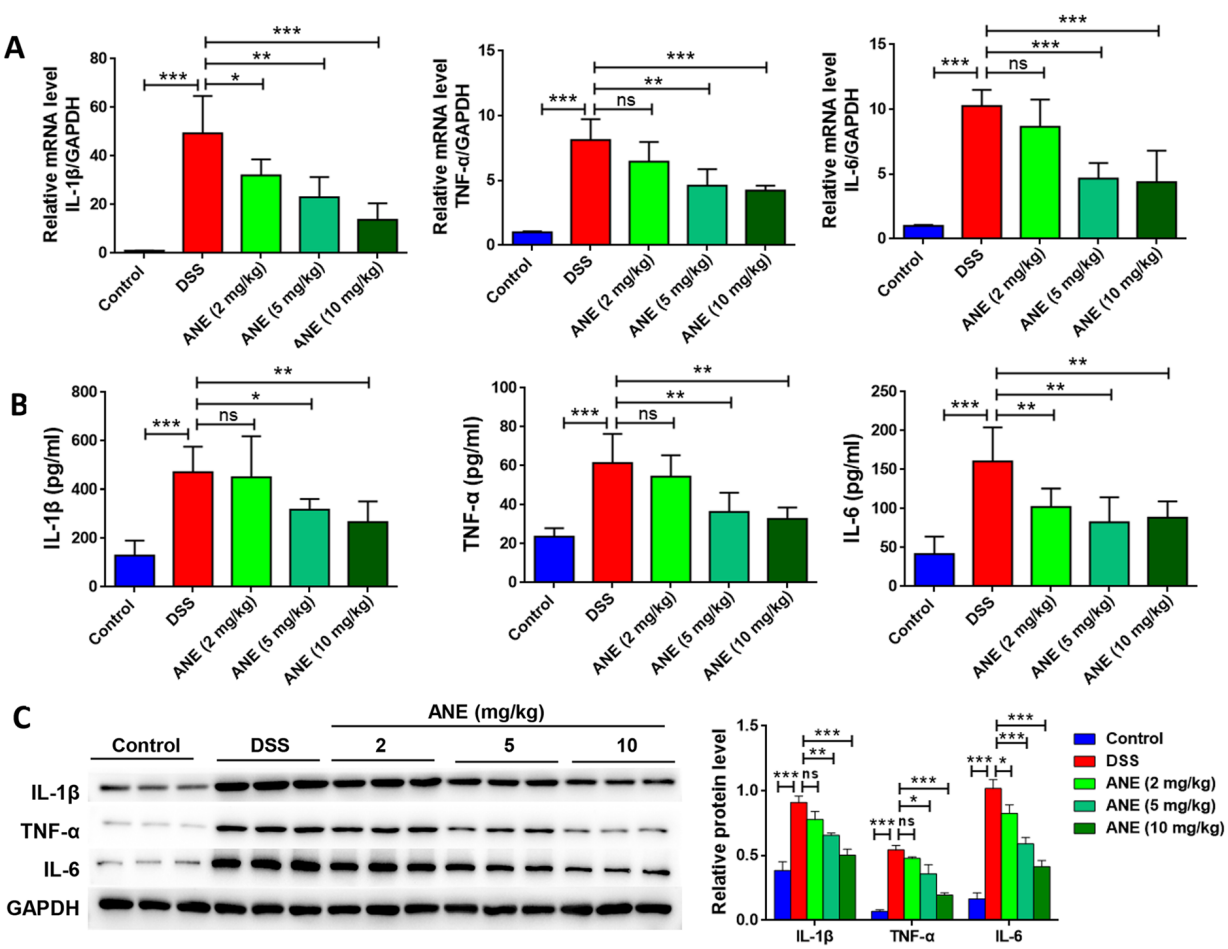
Anemonin improves DSS-induced inflammation in mice. **A** RT-qPCR detects changes in gene transcription levels of inflammatory factors in colon tissue. **B** The secretion of inflammatory factors in colon tissue was detected by ELISA. **C** Protein expression of inflammatory factors in colon tissues was detected by western blotting. *ns* no significance, *p < 0.05, **p < 0.01, ***p < 0.001

To identify the effect of anemonin on tissue inflammation in mice, the expression of IL-1β, TNF-α, and IL-6 in mouse colon tissues was detected using RT-qPCR, ELISA, and western blotting. DSS significantly induced mRNA, protein, and release of IL-1β, TNF-α, and IL-6 in mice (*p* < 0.05, Fig. 2A–C). Anemonin inhibited the production and release of these cytokines in a dose-dependent manner (*p* < 0.05).

### Anemonin attenuates LPS-induced inflammation in HT-29 cells

**Fig. 3:**
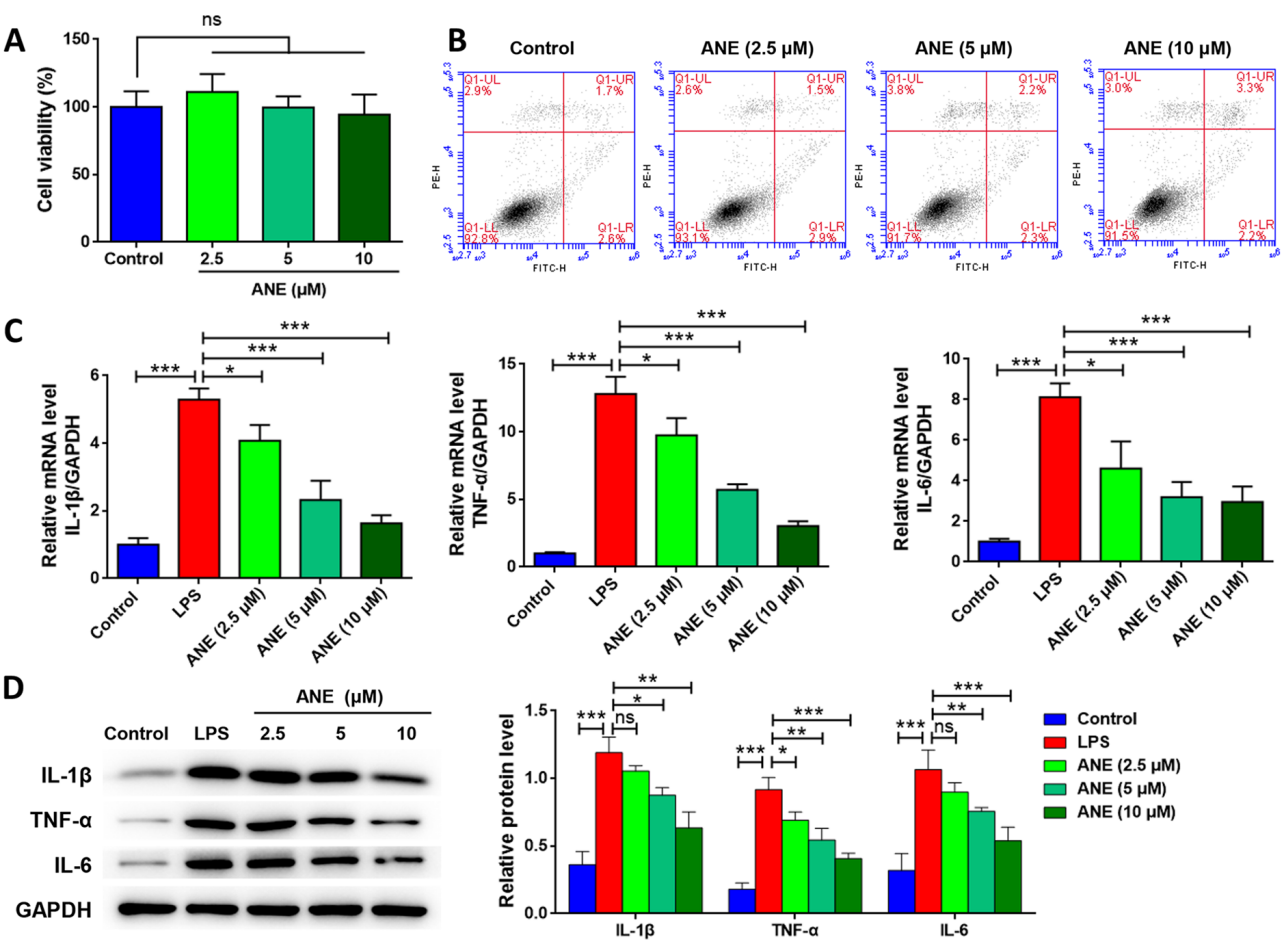
Anemonin attenuates LPS-induced inflammation in HT-29 cells. **A** The viability of HT-29 cells treated with different anemonin concentrations was detected by CCK-8 method. **B** Cell apoptosis was detected by flow cytometry. **C** mRNA levels of inflammatory factors were detected by RT-qPCR. **D** Protein levels of inflammatory factors were detected by western blotting. ns, no significance, *p < 0.05, **p < 0.01, ***p < 0.001

Furthermore, the above results were verified in a cellular inflammation model. HT-29 cells were treated with different anemonin concentrations, cell viability was detected using the CCK-8 assay, and cell apoptosis was detected by flow cytometry (Fig. 3A and B). The results showed that different anemonin concentrations had no significant effect on cell viability or apoptosis, indicating that anemonin was not cytotoxic to HT-29 cells. Next, LPS was used to induce HT-29 cell inflammation. As shown in Fig. 3C and D, LPS significantly upregulates the mRNA and protein levels of IL-1β, TNF-α, and IL-6 (*p* < 0.05), whereas anemonin significantly downregulated these cytokine expression in a dose-dependent manner (*p* < 0.05) (Table 1).

**Table 1:** Primer sequences used for qRT-PCR

	Forward primer	Reverse primer
Mouse IL-1β	5’-TGCCACCTTTTGACAGTGATG-3’	5’-TTCTTGTGACCCTGAGCGAC-3’
Mouse TNF-α	5’-ACCCTCACACTCACAAACCA-3’	5’-GAGGCAACCTGACCACTCTC-3’
Mouse IL-6	5’-GCCTTCTTGGGACTGATGCT-3’	5’-TGTGACTCCAGCTTATCTCTTGG-3’
Mouse GAPDH	5’-GGGGTCCCAGCTTAGGTTCA-3’	5’-CCCAATACGGCCAAATCCGT-3’
Human IL-1β	5’-AGAAGTACCTGAGCTCGCCA-3’	5’-TGGGATCTACACTCTCCAGC-3’
Human TNF-α	5’-AGCCCATGTTGTAGCAAACC-3’	5’-ACATTGGGTCCCCCAGGATA-3’
Human IL-6	5’-TTCGGTCCAGTTGCCTTCTC-3’	5’-TCACCAGGCAAGTCTCCTCA-3’
Human PRKCQ	5’-CACCGGGAAAAAGAGAGCCT-3’	5’-AGTGCTCTGTCGGCAAATGA-3’
Human PTPN2	5’-GAGGAGAACAGTGAGAGTGCT-3’	5’-TGGGCTTCAGGTCTTGCTTT-3’
Human GAPDH	5’-CCATGTTGCAACCGGGAAG-3’	5’-GCCCAATACGACCAAATCAGAG-3’

### PKC-θ is a targeted protein of anemonin

**Fig. 4:**
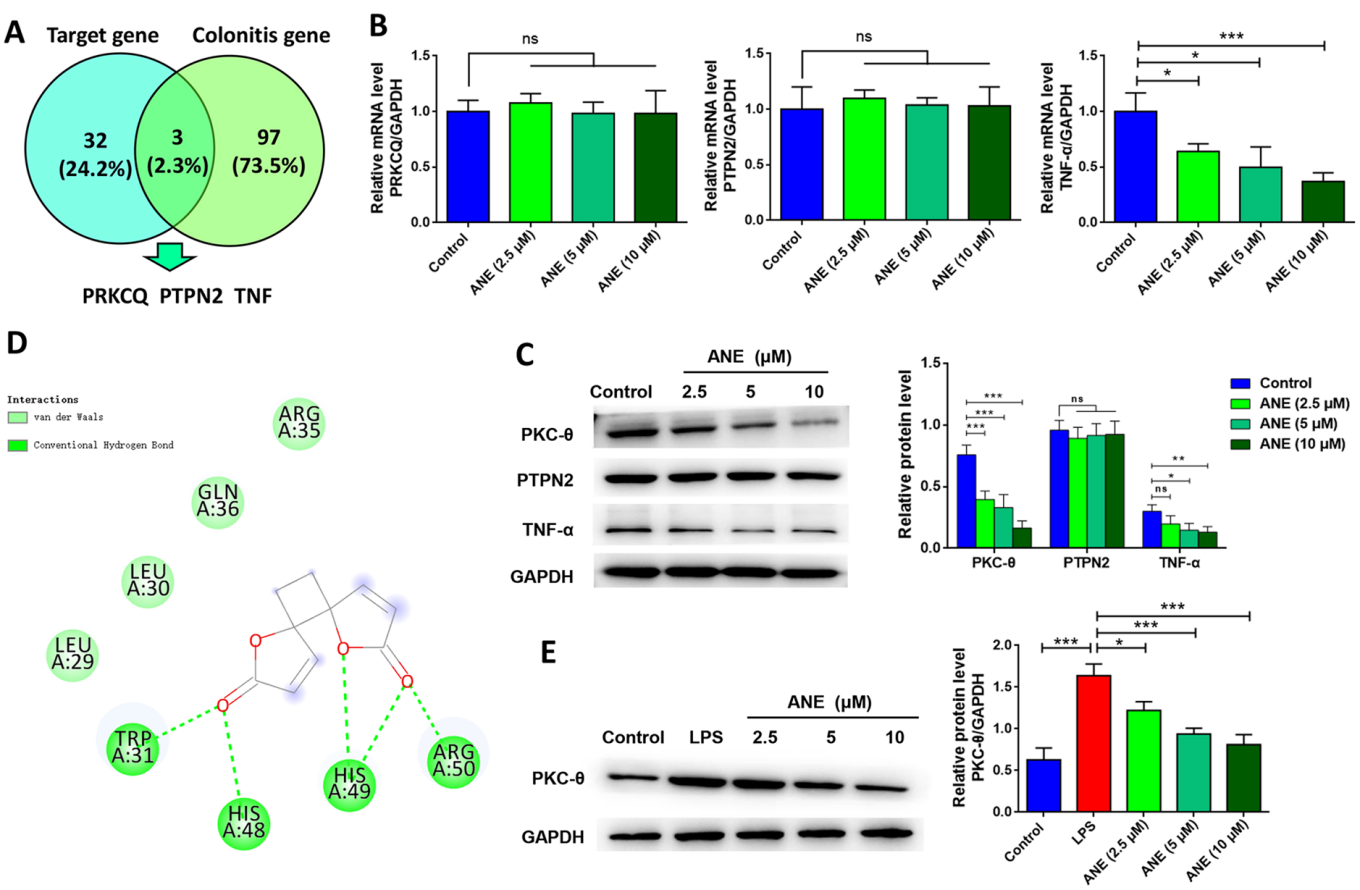
PKC-θ is a target protein of anemonin. **A** Proteins interacting with anemonin were predicted by SwissTargetPrediction, and genes related to enteritis were predicted by CTD website. Three genes were identified by intersection: PRKCQ, PTPN2, and TNF. **B** RT-qPCR was used to detect the transcriptional effects of different concentrations of anemonin on PRKCQ, PTPN2, and TNF-α. **C** Western blotting analysis of the effects of different concentrations of anemonin on PKC-θ, PTPN2, and TNF-α expression. **D** Autodock Vina was used to dock PKC-θ (protein ID: 4FKD) with anemonin (Molecule ID: MOL001883). **E** Western blot was used to determine whether anemonin inhibited PKC-θ expression under LPS-induced conditions. ns, no significance, *p < 0.05, **p < 0.01, ***p < 0.001

Bioinformatic analysis was performed to identify the target proteins of anemonin. SwissTargetPrediction was used to predict the genes that could be targeted by anemonin, and the CTD was used to predict genes related to enteritis. Three genes were identified by intersection: PRKCQ, PTPN2, and TNF (Fig. 4A). The RT-qPCR and western blotting results showed that anemonin did not alter the mRNA or protein levels of PTPN2 (*p* > 0.05, Fig. 4B and C), indicating that PTPN2 is not the target of anemonin. Anemonin significantly inhibited both mRNA and protein levels of TNF-α (*p* < 0.05). However, using AutoDock Vina software, no available molecular docking results were identified between anemonin and TNF-α, indicating that anemonin cannot directly regulate TNF-α expression. Anemonin had no significant effect on PRKCQ mRNA levels (*p* > 0.05), however, it significantly inhibited the protein level of PKC-θ (*p* < 0.05). These results suggest that anemonin does not affect PRKCQ gene transcription but can inhibit PKC-θ protein translation or protein stability. Moreover, anemonin (Molecule ID: MOL001883) was predicted to dock with PKC-θ (protein number: 4FKD) with the binding energy of interaction of -5.9 kcal/mol (Fig. 4D), suggesting that PKC-θ is a direct target of anemonin. Western blot results also showed that anemonin significantly inhibited PKC-θ protein expression under LPS-induced conditions in a dose-dependent manner (*p* < 0.05, Fig. 4E).

### Anemonin attenuates HT-29 cell inflammation via targeted regulation of PKC-θ

**Fig. 5:**
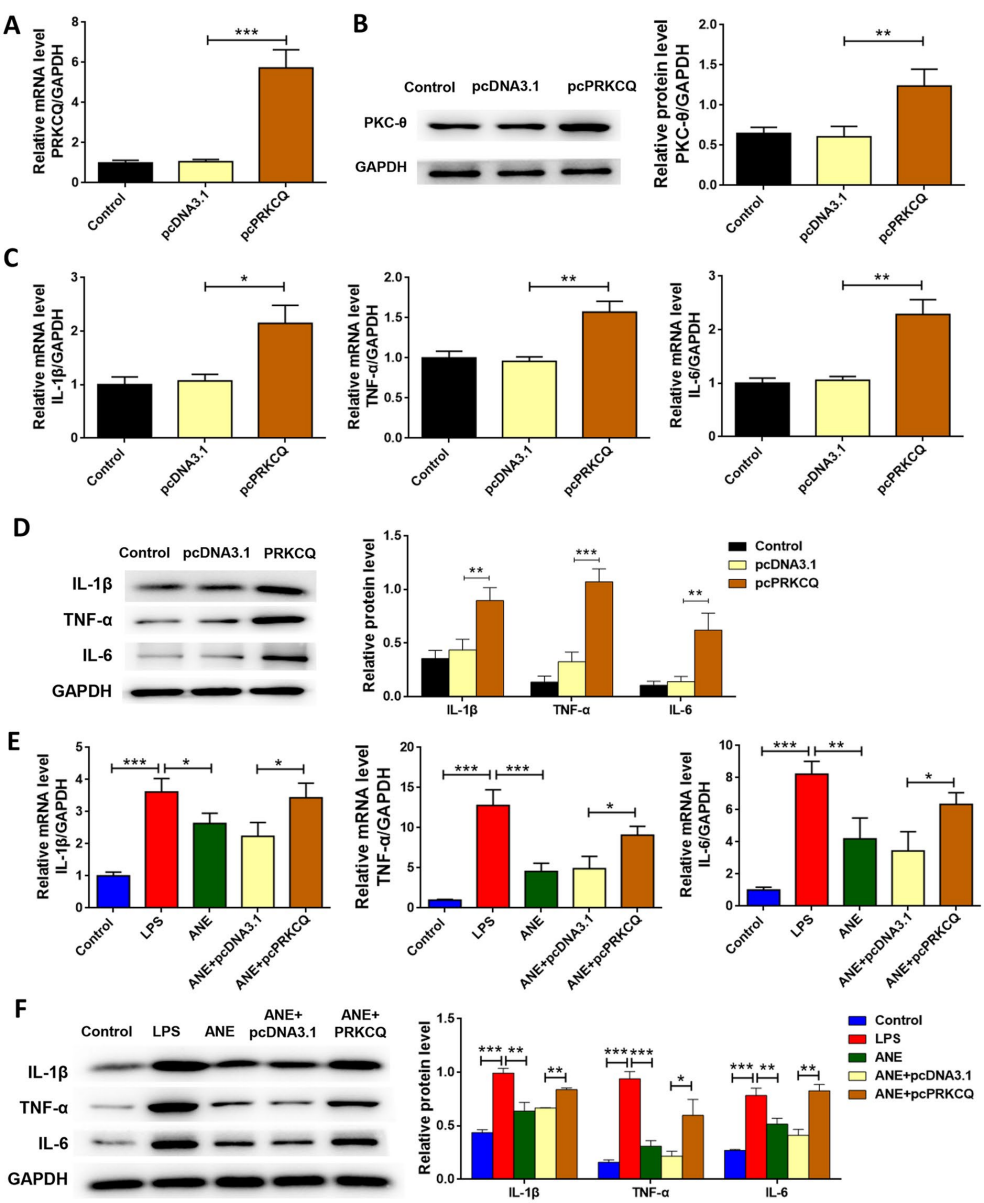
Anemonin attenuates HT-29 cell inflammation via targeted regulation of PKC-θ. **A** RT-qPCR was used to detect whether PRKCQ overexpression plasmid upregulated PRKCQ level. **B** Western blot was used to determine whether PRKCQ overexpression plasmid upregulated PKC-θ protein expression. **C** mRNA levels of inflammatory factors were detected by RT-qPCR, following by transfection with pcPRKCQ or pcDNA3.1. **D** Western blot was used to detect the protein expression levels of inflammatory factors, following by transfection with pcPRKCQ or pcDNA3.1. **E** mRNA levels of inflammatory factors were detected by RT-qPCR, following by the transfected HT-29 cells stimulated with LPS with or without anemonin treatment. **F** Western blot was used to detect the protein expression levels of inflammatory factors, following by the transfected HT-29 cells stimulated with LPS with or without anemonin treatment. *p < 0.05, **p < 0.01, ***p < 0.001

To evaluate whether anemonin protects HT-29 cells via PKC-θ regulation, a PRKCQ overexpression plasmid (pcPRKCQ) was transfected into HT-29 cells. Figure 5A and 5B show that, cell transfection with pcPRKCQ significantly increases the PRKCQ mRNA and PKC-θ protein levels (*p* < 0.05). The mRNA and protein levels of IL-1β, TNF-α, and IL-6 were significantly increased in cells transfected with pcPRKCQ (*p* < 0.05, Fig. 5C and 5D) compared with that of the cells transfected with the empty pcDNA3.1 plasmid. In addition, cell transfection with pcPRKCQ significantly reversed the inhibitory effects of anemonin on cytokine production (*p* < 0.05, Fig. 5E and F). The anti-inflammatory effects of anemonin on HT-29 cells appeared to be reversed by PRKCQ overexpression.

### Anemonin attenuates DSS-induced colitis in a murine model via targeted PKC-θ regulation

**Fig. 6:**
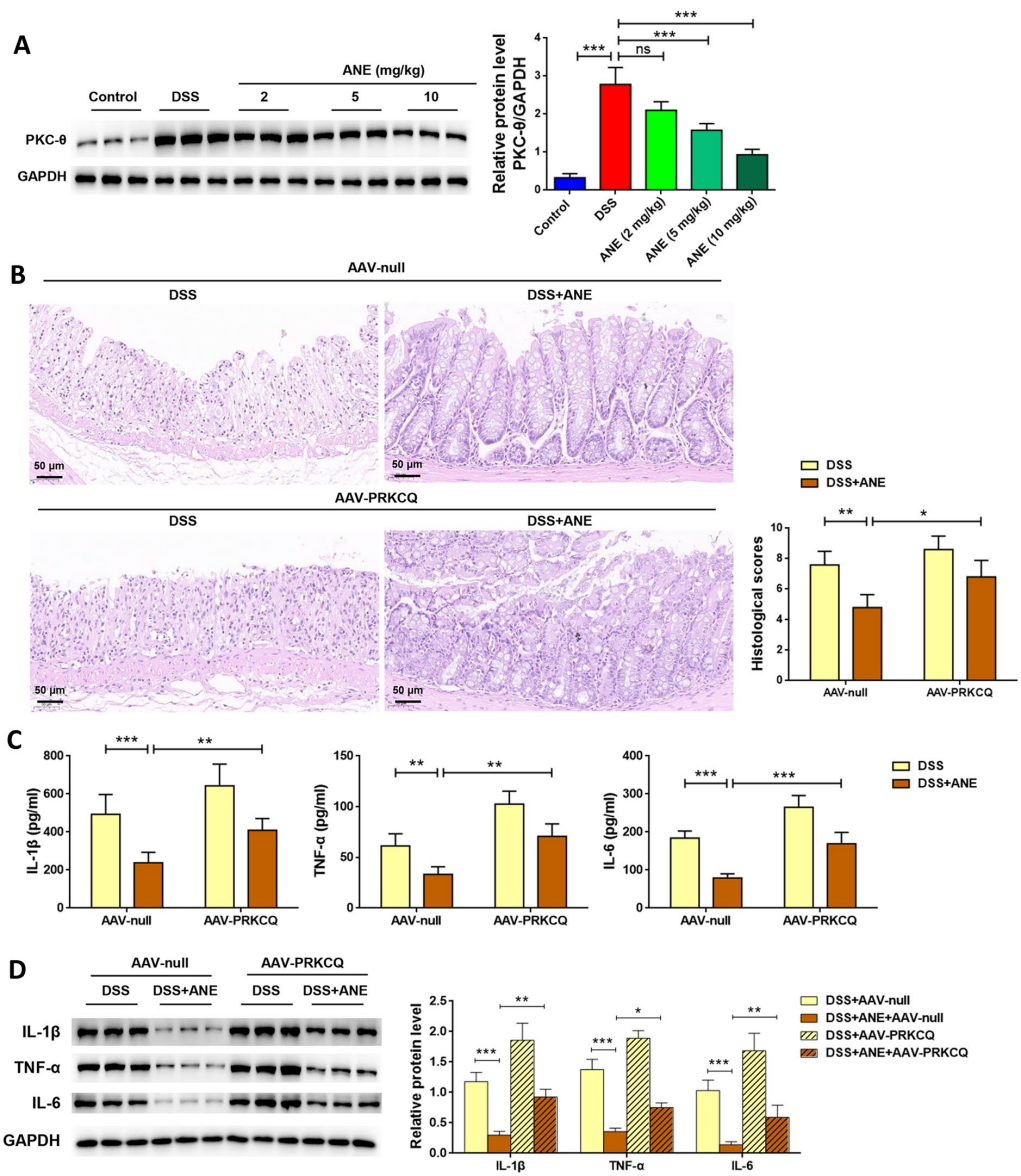
Anemonin attenuates DSS-induced colitis in a murine model via targeted PKC-θ regulation. **A** Protein expression of PKC-θ in colon tissues was detected by western blotting. **B** Colonic tissues of mice were stained with HE staining to evaluate the degree of tissue damage. **C** The secretion of inflammatory factors in colon tissue was detected by ELISA. **D** Protein expression of inflammatory factors in colon tissues was detected by western blot. *p < 0.05, **p < 0.01, ***p < 0.001

The regulation of PKC-θ expression by anemonin was then confirmed in vivo. Compared with that of control mice, PKC-θ expression in the colon tissues of DSS-induced mice was significantly increased (*p* < 0.05, Fig. 6A). Administration of anemonin significantly inhibited PKC-θ expression in a dose-dependent manner (*p* < 0.05). To further confirm the anti-inflammatory effects of anemonin via the regulation of PKC-θ, AAV delivery of PRKCQ-expressing vectors was instilled into the mouse colon one week before DSS induction. Compared with those of the AAV-null group, mice that received AAV-PRKCQ had significantly higher histological scores (*p* < 0.05, Fig. 6B). Moreover, compared with those of AAV-null mice, mice that received AAV-PRKCQ had higher levels of IL-1β, TNF-α, and IL-6 in the colon tissues (*p* < 0.05, Fig. 6C and D).

## Discussion

In this study, the pharmacological effects of anemonin on acute UC were investigated, and it was determined that anemonin exerts anti-inflammatory effects by inhibition of PKC-θ protein expression. In this study, a mouse model of UC was established using DSS. Anemonin can alleviate weight loss, colon length shortening, DAI score increases, colon tissue damage, and colon inflammation caused by DSS. The intestinal epithelial cell line HT-29 was treated with LPS to construct a cell model. Anemonin was found to have no cytotoxic effect on HT-29 cells, however, it significantly alleviated LPS-induced inflammation. PKC-θ was determined to be a target protein of anemonin. Anemonin did not affect PRKCQ gene transcription but significantly inhibited PKC-θ protein translation or protein stability. Moreover, in vitro and in vivo data confirmed the anti-UC effects of anemonin via targeted PKC-θ regulation. This study provides a theoretical basis for the clinical application of anemonin in UC treatment.

The occurrence of UC is closely related to immune function, intestinal microbes, genetic factors, and environmental factors, among which immune dysfunction plays an important role in the occurrence and development of UC [7]. There is a proportional relationship between histological inflammation and colorectal cancer risk [22], therefore, this study is essential. In this study, DSS was used to induce an acute UC model, and LPS was used to treat HT-29 cells to construct cell inflammation models. Both are widely accepted pathological models with stable and reliable results [23, 24]. A variety of bioinformatics methods were used in order to identify the target protein of anemonin. SwissTargetPrediction allows us to estimate the most probable macromolecular targets of a small molecule [25, 26]. The CTD was used to screen for genes associated with UC [27]. For further confirmation, AutoDock Vina, a widely cited open-source molecular docking software [28] was used, and the final docking results suggested that PKC-θ might be the target protein of anemonin. Subsequent cell experiments verified the predicted results at an experimental level.

Anemonin is a pentacyclic triterpenoid exists in various Ranunculaceae and Gramineae plants [29]. Anemonin has been reported to have various pharmacological activities, especially the anti-inflammatory activity [30]. For instance, administering mice with anemonin attenuated osteoarthritis progression via inhibiting IL-1β activation [31]. Anemonin has benefit in attenuating intestinal inflammation by inhibition of LPS-induced intestinal microvascular endothelial cell damage and nitric oxide release [32]. Dietary anemonin improved intestinal barrier restoration and protected piglets against LPS-induced intestinal inflammation [33]. In line with these previous findings, we confirmed the anti-inflammatory activities of anemonin in mice colon, as evidenced by the observed downregulation of IL-1β, TNF-α, and IL-6. Anemonin shows potential in the treatment of UC.

PKC-θ is a serine/threonine kinase that belonging to the PKC subfamily [34]. In the context of the immune system, PKC-θ dysfunction leads to autoimmune and inflammatory diseases [35]. PKC-θ is highly expressed in T lymphocytes, and PKC-θ-deficient mice have been used to elucidate the role of PKC-θ in the T cell immune response [36, 37]. PKC-θ is critical for T helper (Th)2- and Th17-mediated responses^35^, and certain specific Th1 responses are altered in PKC-θ-deficient mice [38]. PKC-θ is highly expressed and activated in immunological disorders. PKC-θ inhibitors showed encouraging results in the context of immunosuppressive therapy for autoimmune diseases such as psoriasis [39]. Another study showed that missense mutations in PRKCQ are associated with Crohn's disease [40]. Studies have shown that PKC-θ plays a fundamental role in different types of chronic colitis [41]. In this study, PKC-θ was found to be a target protein of anemonin by regulating of which anemonin exerted its anti-UC properties. This result provides a deep understanding of the mechanism by which anemonin protects colon tissues against inflammatory responses.

## Conclusions

In conclusion, anemonin relieved the symptoms and inflammatory responses in a mouse model of DSS-induced UC. Anemonin inhibits the expression of inflammatory cytokines in cellular inflammatory models. This process can be achieved by targeting the PKC-θ.

## Data Availability

The datasets used and/or analysed during the current study available from the corresponding author on reasonable request.

## Abbreviations

UC: Ulcerative colitis
DSS: Dextran sulfate sodium
IBD: Inflammatory bowel disease
